# Expression levels of uridine 5'-diphospho-glucuronosyltransferase genes in breast tissue from healthy women are associated with mammographic density

**DOI:** 10.1186/bcr2632

**Published:** 2010-08-27

**Authors:** Vilde D Haakensen, Margarethe Biong, Ole Christian Lingjærde, Marit Muri Holmen, Jan Ole Frantzen, Ying Chen, Dina Navjord, Linda Romundstad, Torben Lüders, Ida K Bukholm, Hiroko K Solvang, Vessela N Kristensen, Giske Ursin, Anne-Lise Børresen-Dale, Åslaug Helland

**Affiliations:** 1 Department of Genetics, Institute for Cancer Research, Oslo University Hospital Radiumhospitalet, Oslo, Montebello, NO-0310, Norway; 2 Institute for Clinical Medicine, Faculty of Medicine, University of Oslo, Oslo, NO-0315, Norway; 3 Biomedical Research Group, Department of Informatics, University of Oslo, Oslo, NO-0315, Norway; 4 Centre for Cancer Biomedicine, University of Oslo, Oslo, NO-0315, Norway; 5 Department of Radiology, Oslo University Hospital Radiumhospitalet, Oslo, NO-0310, Norway; 6 Department of Radiology, University Hospital of North Norway, Tromsø, NO- 9038, Norway; 7 Department. of Pathology, Vestfold Hospital, Halfdan Wilhelmsens Alle' 17, Tønsberg, NO-3103, Norway; 8 Department of Radiology, Innlandet Hospital, Brummundal, NO-2381, Norway; 9 Department of Radiology, Buskerud Hospital, Drammen, NO-3004, Norway; 10 Department for Clinical Molecular Biology (EpiGen), Institute for Clinical Medicine, Akershus University Hospital, University of Oslo, Oslo, NO-0315, Norway; 11 Department of Surgery, Akerhus University Hospital, Lørenskog, 1478, Norway; 12 Department of Nutrition, School of Medicine, University of Oslo, Oslo, NO-0315, Norway; 13 Department of Preventive Medicine, University of Southern California Keck School of Medicine, Los Angeles, CA 90033, USA; 14 Department of Oncology, Oslo University Hospital Radiumhospitalet, Oslo, NO-0310, Norway

## Abstract

**Introduction:**

Mammographic density (MD), as assessed from film screen mammograms, is determined by the relative content of adipose, connective and epithelial tissue in the female breast. In epidemiological studies, a high percentage of MD confers a four to six fold risk elevation of developing breast cancer, even after adjustment for other known breast cancer risk factors. However, the biologic correlates of density are little known.

**Methods:**

Gene expression analysis using whole genome arrays was performed on breast biopsies from 143 women; 79 women with no malignancy (healthy women) and 64 newly diagnosed breast cancer patients, both included from mammographic centres. Percent MD was determined using a previously validated, computerized method on scanned mammograms. Significance analysis of microarrays (SAM) was performed to identify genes influencing MD and a linear regression model was used to assess the independent contribution from different variables to MD.

**Results:**

SAM-analysis identified 24 genes differentially expressed between samples from breasts with high and low MD. These genes included three uridine 5'-diphospho-glucuronosyltransferase (*UGT*) genes and the oestrogen receptor gene (*ESR1*). These genes were down-regulated in samples with high MD compared to those with low MD. The UGT gene products, which are known to inactivate oestrogen metabolites, were also down-regulated in tumour samples compared to samples from healthy individuals. Several single nucleotide polymorphisms (SNPs) in the *UGT *genes associated with the expression of *UGT *and other genes in their vicinity were identified.

**Conclusions:**

Three UGT enzymes were lower expressed both in breast tissue biopsies from healthy women with high MD and in biopsies from newly diagnosed breast cancers. The association was strongest amongst young women and women using hormonal therapy. UGT2B10 predicts MD independently of age, hormone therapy and parity. Our results indicate that down-regulation of *UGT *genes in women exposed to female sex hormones is associated with high MD and might increase the risk of breast cancer.

## Introduction

Breast cancer is a common disease in women. Knowledge about the first steps in tumour initiation is important for early detection. However, the exact mechanisms of tumour initiation are still unknown.

Mammographic density (MD), captured on film screen mammograms, refers to the content and architectural structure of the adipose, connective and epithelial tissues in the female breast [1]. In epidemiological studies, a high percentage of MD confers a four to six fold elevated risk of developing breast cancer [1-3] and has been proposed as a possible surrogate marker for the disease [4]. The relative risk associated with MDs remains at this magnitude even after adjustment for all other known breast cancer risk factors. Breasts with high MD have greater tissue cellularity and more tissue collagen [5]. Still, little is known as to how MD confers the increased breast cancer risk. MD is to a large degree an inherited trait, although it is also influenced by environmental factors, hormone therapy being an evident example [6]. The genetic factors determining the inheritability are largely unknown.

In order to elucidate how MD increases the risk of breast cancer; we searched for the biological correlates to MD. Gene expression analysis on biopsies from breasts of healthy women with varying degrees of MD was performed. The gene expression profiles represent the gene activity of the different cell types in the biopsy, producing a fingerprint of the breast tissue within the biopsy of that particular woman.

**Figure 1:**
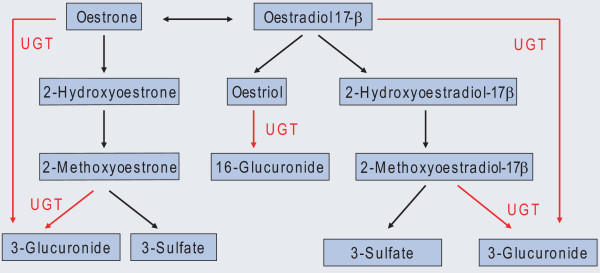
**UGTs conjugate oestrogen-substrates into biologically inactive oestrogen glucuronides**. The figure gives a schematic view with focus on glucuronidation and not a complete picture of oestradiol metabolism. Androgens are also inactivated by uridine 5'-diphospho-glucuronosyltransferases (UGTs), but are not included in this illustration.

The breast is an oestrogen-sensitive organ. MD varies with levels of female hormones, and is reduced after menopause. The uridine 5'-diphospho-glucuronosyltransferase (*UGT*) genes encode enzymes inactivating several endogenous and exogenous compounds, including sex hormones (Figure 1) [7]. UGT1A1 is known to be responsible for the glucuronidation of bilirubin, but is also shown to glucuronidate catechol oestrogens [8,9]. Polymorphisms in this gene have previously been linked to MD in premenopausal women [10]. UGT2B7 is known to conjugate oestrone, one of the active oestradiol metabolites. This enzyme has previously been found to be down-regulated in tumour tissue compared with non-malignant tissue, leading to the conclusion that UGT expression could lead to the promotion of carcinogenesis [11] but there are no reports on this gene in relation to MD in the literature. Less is known about the other *UGT2B *genes, although there is extensive structural homology. We will use the *UGT *genes as a term describing three *UGT2B *genes significantly down-regulated in our analyses (*UGT2B7*, *UGT2B10 *and *UGT2B11*). Other *UGT *genes are specified in the text. In this study we analysed biopsies from breasts of healthy women and found genes whose expression is associated with MD.

## Materials and methods

### Subjects

The women included in this study had all attended one of six breast diagnostic centres in Norway that are part of the governmentally funded National Breast Cancer Screening Program between 2002 and 2007 [12]. Women were eligible if they did not currently use anticoagulants, did not have breast implants and were not currently pregnant or lactating. A total of 186 women were recruited to the study; 120 healthy women with no malignant disease but some visible density in the mammograms, referred to here as healthy women, and 66 women with a newly diagnosed breast cancer. Of these, quality tested expression data were obtained from biopsies from 79 healthy women and 64 breast cancer patients.

The women were either referred to a breast diagnostic centre for a second look due to some irregularity of the initial screening mammogram (n = 69) or due to clinical findings (n = 83). For 34 women the type of referral was unknown.

The women provided information about height, weight, parity, hormone therapy use and family history of breast cancer. Two breast biopsies and three blood samples were collected from each woman. All women provided signed informed consent. The study was approved by the local ethical committee and local authorities (IRB approval no S-02036).

### Core biopsies

Two breast biopsies were obtained from each woman with a 14 gauge needle, for RNA- and DNA-extraction. In healthy women, the biopsies were taken from an area with no visible pathology, but with some MD to ensure that the biopsies did not contain only fatty tissue, which yields little RNA. The sampling was guided by ultrasound. At one hospital, six of the biopsies from breasts of healthy women were collected from a benign lesion (mostly fibroadenomas). For the cancer patients, all biopsies were taken from the tumour. The tissue was either fresh-frozen at -80°C or soaked in ethanol and RNAlater (Ambion, Austin, TX, USA), transported and subsequently stored at -20°C.

### Pathology

The haematoxylin eosinofil sections from the tumours of the breast cancer patients were evaluated locally and then re-evaluated by one pathologist (YC). Information about tumour size, histological grade and type, oestrogen and progesterone receptor status, human epidermal growth factor receptor (HER) 2 status and sentinel node status was recorded and entered into a database managed by the Office for Clinical Research at Oslo University Hospital, Radiumhospitalet. Pathology evaluations were not available for the biopsies from breasts of healthy women.

### RNA-expression analysis

Homogenisation, cell lysis and RNA extraction were performed using the RNeasy Mini Protocol (Qiagen, Valencia, CA, USA). RNA quality was controlled by Agilent 2100 Bioanalyzer (Agilent Technologies, Palo Alto, CA, USA) and concentration was determined using NanoDrop ND-1000 spectrophotometer (Thermo Scientific, Wilmington, DE, USA). A total of 40 samples, mostly from normal breast tissue, were excluded from further analyses due to a low RNA amount (< 10 ng) or poor RNA quality. RNA was then amplified and labelled using the Agilent Low RNA input Fluorescent Linear Amplification Kit Protocol. Amplified tumour RNA was labelled by Cy5 (Amersham Biosciences, Little Chalfont, England, UK) and amplified RNA from Universal Human total RNA (Stratagene, La Jolla, CA, USA) was labelled by Cy3 (Amersham Biosciences, Little Chalfont, England, UK). RNA from the remaining 146 biopsies was further hybridised on Agilent Human Whole Genome Oligo Microarrays (G4110A) (Agilent Technologies, Santa Clara, CA, USA). Three arrays had to be excluded due to poor quality leaving data from 143 subjects (79 healthy individuals and 64 breast cancer patients) for further analysis. Of the 79 biopsies from healthy women, 5 had been obtained from a benign lesion. By ultrasound and mammography these 5 were described as fibroadenoma (n = 4) or microcalcification (n = 1).

### RNA-data processing

The microarrays were scanned by an Agilent scanner (Agilent Technologies, Santa Clara, CA, USA) and processed in Feature Extraction 9.1.3.1 (Agilent Technologies, Santa Clara, CA, USA). Locally weighted scatterplot smoothing (lowess) was used to normalise the data. The normalised and log2-transformed data were stored in the Stanford Microarray Database [13] and retrieved from the database for further statistical analyses. Flagged spots were treated as missing values. The dataset now counted 40,791 probes. Clone IDs with 20% or more missing values were excluded. Gene filtering was performed to include only probes with variation across samples, so that probes with less than three arrays being at least 1.6 standard deviations from the mean were excluded. For the 79 healthy women, this probe filtration resulted in an expression dataset of 9,767 probes and 79 arrays each representing one individual. For the breast cancer women, a dataset of 64 arrays and 10,153 probes were obtained after filtration, and for both groups combined, a dataset of 143 arrays and 13,699 probes were obtained. Missing values were imputed in R using the method impute.knn in the library impute [14].

### Genotyping

Blood DNA was extracted by phenol/chloroform extraction followed by ethanol precipitation (Nuclear Acid Extractor 340A; Applied Biosystems, Foster City, CA, USA) according to standard procedures. *UGT *genotype data was retrieved from two sources: genome wide association studies (GWAS) using the Human-1 109K BeadChip (Illumina Inc, San Diego, CA, USA) and candidate gene-based study using iPlex, Sequenom. For the GWAS, each sample was subject to whole genome amplification using Illumina proprietary reagents [15]. The amplified DNA was fragmented and hybridised according to the protocol. The BeadArray reader (Illumina Inc, San Diego, CA, USA) with the BeadScan software (Illumina Inc, San Diego, CA, USA) was used to image the beadchips. Non-polymorphic probes and probes with more than 20% missing values and were excluded and data processed as described previously [16]. The candidate gene single nucleotide polymorphism (SNP) analyses were performed using the iPLEX assay in conjunction with the Sequenom MassARRAY platform. Multiplexing was performed in 384 plates using 1 ul DNA per well with one well containing up to 29 reactions. The technology is described in detail on the sequenom web-page [17].

### Mammograms

Routine descriptions of mammograms by local radiologists were collected. Craniocaudal mammograms of both breasts were digitised using a high-resolution Kodak Lumisys 85 scanner (Kodak, Rochester, NY, USA). Density was quantified using the University of Southern California Madena assessment method [18]. In brief, the method works as follows: a reader (trained by GU) outlines the total area of the breast using a computerised tool, the software then counts the number of pixels. This represents the total breast area. MD is assessed (by GU), first by identifying a region of interest that incorporates all dense areas except those representing the pectoralis muscle and scanning artifacts, and then by applying a yellow tint to all pixels within the region of interest shaded at or above a threshold intensity of gray. The software then counts the tinted pixels, which represents the area of absolute density. The percent density is the absolute density area divided by the total breast area and is the value used for these analyses. Test-retest reliability was 0.99 for absolute density.

### Statistical analysis

Clustering was performed using MatLab (version R2007b) (The MathWorks Inc., Natick, MA, USA) with Ward linkage and Euclidean distances. Before clustering, the data were gene centred, that is, for every probe the mean expression across all samples was calculated and was subtracted from the log2-ratios for that gene. This was performed for visualisation purposes only, clustering with uncentred data returns the same clusters. Significance analysis of microarrays (SAM, Stanford University, CA, USA) (version 3.02) [19,20] for Excel (Microsoft, Redmond, WA, USA) was used for analysis of differentially expressed genes between two groups of data. The data were not gene centred for the SAM analysis. A total of 500 permutations were used. Quantitative SAM analysis was used to identify genes differentially expressed according to MD as a continuous variable. Statistical significance tests and regression analysis were performed in R 2.9.0 [21]. To test for difference in the mean of phenotypic variables (MD, age, body mass index (BMI)) in different clusters of women, we used two-sided t-tests (assuming equal variance in the groups) and analysis of variance (ANOVA) for continuous variables and chi-squared/Fisher's exact tests for categorical variables [22]. To investigate the similarities of distributions of *UGT *genes between tumour samples and normal samples with low MD and high MD respectively, Kullback-Leibler distances between normalised distributions of the histograms of the data were calculated by use of MatLab (The MathWorks Inc., Natick, MA, USA). The cancer samples in our study were grouped into subtypes and assigned a risk group using the PAM50 gene list published by Parker et al [23]. SNP-analysis was performed using R 2.9.0 [21]. The association between gene expression and SNPs was assessed using expression quantitative trait loci (eQTL) [24]*in cis *(10^6 ^bp on each side of the gene) using the R package eMap v1.1 [25]. Comparing the akaike information criterion for different models predicting MD, the lower criterion singled out a linear regression model as the model fitting the distribution of the data best. A linear regression model was fitted in R 2.9.0 with MD as a continuous response variable and the following covariates: UGT2B7, two probes for UGT2B10, UGT2B11, ESR1, age, BMI, current hormone therapy, age at first birth and parity. Gene expression, age, age at first birth and BMI were entered into the model as continuous variables. Stepwise variable selection was performed, starting with all variables included in the model. For every step, the variable with the highest *P *value was rejected from the model and the model was refitted. This was repeated until all variables included in the model had a *P *value less than 0.05. To correct for the influence of age, this variable was forced to stay in the model. A sensitivity analysis was performed excluding extreme ages (30 years or younger) to check the robustness of the data. We also fitted linear regression stratified on age (younger or older than 50 years of age) and current use of hormone therapy. Gene ontology analysis was performed by the use of DAVID Bioinformatics Resources 2008 from the National Institute of Allergy and Infectious Diseases, NIH [26]. Functional annotation clustering was applied and the following gene ontology categories were selected: biological processes (all), molecular function (all) and the KEGG pathway database. We included gene ontology terms with a *P *value (false discovery rate (FDR)-corrected) of less than 0.01 containing between 5 and 500 genes.

The normalised, log2-transformed data are available in Gene Expression Omnibus with accession number [GEO:GSE18672]. The data are not gene centered or gene filtered.

## Results

### Gene expression and mammographic density

To identify genes differentially expressed according to MD we performed quantitative SAM with MD as a continuous variable using gene expression data from the normal biopsies. Of 9,767 probes, only 25 probes, representing 24 genes, were differentially expressed according to MD, with reduced expression associated with higher MD (FDR < 25%; Table 1) [see Additional file 1]. Gene ontology analysis revealed no significant terms and we found no pathway associated with this gene set. The *UGT *genes and oestrogen receptor gene (*ESR1*) were among the genes significantly down-regulated in breasts with high MD. The percentage of samples with low UGT expression was higher in tumour samples than in normal samples with low MD, whereas the percentage was more similar between tumour samples and normal samples with high MD [see Figure S1 in Additional file 2]. The function of UGT-enzymes in oestradiol metabolism is illustrated in Figure 1. In healthy women, the expression of the different *UGT *genes was highly correlated with each other and the four probes clustered together [see Figures S2 and S3 and Table S1 in Additional file 2].

**Table 1:** Genes differentially expressed according to mammographic density in non-cancer samples

Gene symbol	Agilent ID	Cytogenetic band
729641	A_24_P932736	8p21.1
FLJ10404	A_23_P427472	5q35.3
VPS18	A_24_P18802	15q15.1
UGT2B10	A_23_P7342	4q13.2
CABP7	A_24_P177236	22q12.2
CD86	A_24_P131589	3q13.33
UGT2B11	A_23_P212968	4q13.2
580687	A_23_P152570	17p11.2
DIAPH2::RPA4	A_23_P254212	Xq21.33
LMOD1	A_32_P199824	1q32.1
UGT2B10	A_24_P521559	4q13.2
PIK3R5	A_23_P66543	17p13.1
ATG7	A_32_P107994	3p25.2
LRRC2	A_23_P155463	3p21.31
RBL1	A_23_P28733	20q11.23
NPY1R	A_23_P69699	4q32.2
810781	A_23_P144244	3q13.33
593535	A_32_P80016	15q26.1
H2AFJ	A_23_P204277	12p12.3
666399	A_32_P35668	20p12.3
Transcribed	A_24_P640617	2p25.2
Transcribed	A_32_P20997	20q13.13
UGT2B7	A_23_P136671	4q13
ESR1	A_23_P309739	6q25.1
SAPS1	A_23_P119448	19q13.42

MD was lower in women with BMI of 25 or more compared with those with BMI of less than 25 (*P *= 0.01), but unrelated to other epidemiological variables. UGT expression was not significantly associated with age, BMI, age at first birth or current hormone therapy use in the healthy women [see Table S2 in Additional file 2].

To dissect the impact of age and hormone therapy use, we performed SAM analyses to identify differentially expressed genes according to MD, whereas stratifying for age and postmenopausal hormone therapy use. For healthy women younger than 50 years of age, the *UGT *genes were not significant at a FDR of 25%. For healthy women aged 50 years or older, 49 probes were significantly down-regulated in breasts with MD of 30% or higher (FDR < 25%). Of these, 17 were overlapping with those significantly down-regulated among healthy women in the unstratified analysis. The *UGT *genes were not in this list. We then stratified the women aged 50 years or older on current hormone therapy use. When only those currently using hormone therapy were included in the analysis, *UGT2B7 *and *UGT2B11 *were among the six genes differentially expressed with an FDR less than 10E-5 and *UGT2B28 *with FDR less than 25%. For healthy women above 50 years of age and not currently using hormone therapy, several of the 24 genes were differentially expressed according to MD with an FDR of less than 25%, but again the *UGT *genes were not in this list [see Additional file 3].

These analyses were confirmed fitting a linear regression model. Although the other variables were excluded from the model with insignificant *P *values, age was kept in the model to control for the age-effect. After stepwise variable selection, the only significant variables remaining in the model were UGT2B10 (A_23_P7342)(*P *= 0.005) and BMI (*P *= 0.015). Sensitivity analysis excluding extreme ages (30 years and younger) did not alter the results (UGT2B10 *P *= 0.003, BMI *P *= 0.016) and indicates the robustness of the results. ESR1 was borderline significant in both these analyses. These results were not significantly altered when MD was log2-transformed. For further stratification see Table 2.

**Table 2:** Linear regression analysis of factors predicting mammographic density in all women and stratified for age and hormone therapy use

Women in model	N	Variables	Beta value	*P *value
All women	76	UGT2B10^1)^	-0.6	0.902
		UGT2B7	1.8	0.631
		UGT2B11	4.8	0.275
		ESR1	-3.8	0.055
		**UGT2B10**^2)^	**-5.6**	**0.005**
		**BMI**	**-1.5**	**0.015**
		age	-0.4	0.074
50 years or older	46	UGT2B11	0.2	0.987
		UGT2B10^1)^	1.0	0.946
		UGT2B7	3.5	0.486
		UGT2B10^2)^	-3.7	0.073
		BMI	-1.4	0.052
		**ESR1**	**-6.0**	**0.016**
		age	-0.9	0.061
50 years or older, currently on hormone therapy	11	UGT2B10^1)^	7.2	0.771
		UGT2B11	-5.8	0.695
		BMI	-2.9	0.103
		UGT2B7	6.8	0.418
		**UGT2B10^2)^**	**-27.0**	**0.000**
		**ESR1**	**-8.1**	**0.011**
		age	-0.9	0.103
50 years or older, never used hormone therapy	28	UGT2B11	-0.7	0.948
		UGT2B10^1)^	3.3	0.809
		UGT2B7	3.1	0.555
		UGT2B10^2)^	-1.4	0.607
		BMI	-0.9	0.348
		**ESR1**	**-6.0**	**0.033**
		**Age**	**-1.5**	**0.004**
Younger than 50 years	30	UGT2B7	0.4	0.950
		UGT2B10^1)^	-1.2	0.866
		ESR1	-0.9	0.835
		UGT2B11	8.4	0.225
		BMI	-1.4	0.216
		**UGT2B10^2)^**	**-6.2**	**0.040**
		Age	-0.3	0.610

1) A_24_P521559, 2) A_23_P7342

Factors predicting mammographic density (MD) after stepwise exclusion of non-significant factors are shown. Variables listed in the order of exclusion from the model. *P *value from the last equation including the variable is shown. Age is forced to stay in the model. UGT2B10 (A_23_P7342) is a significant, independent predictor of MD in all analyses with a majority of women under influence of female hormones; women younger than 50 years of age and women currently on hormone therapy. BMI, body mass index.

Unsupervised hierarchical clustering of the 79 samples from healthy women showed two main clusters. MD was not significantly different between these two clusters [see Figure S3 in Additional file 2].

In the breast cancer group, MD was significantly associated with age and BMI, with higher MD in the younger women and in those with BMI less than 25. Both MD and UGT expression tended to be higher in women with receptor positive tumours, but this was not significant for any type of receptor. UGT-expression in tumours was unrelated to age, BMI, age at first birth and current hormone therapy (data not shown). There was a higher proportion of oestrogen receptor positive tumours among the breast cancer patients with high MD (≥ 30%) compared with low (< 30%) MD (10 of 10 vs 36 of 40, Fisher's = 0.001). There was no significant association between tumour subtype and level of MD as assessed by ANOVA. There was no indication that degree of MD was associated with the risk of relapse as assessed by the method of Parker et al [23] [see Figure S4 of Additional file 2].

Nine probes were differentially expressed according to MD in cancer samples (FDR < 25%; Table 3). None of these were overlapping with the 24 genes differentially expressed in the samples from the breasts of healthy women.

**Table 3:** Genes differentially expressed according to mammographic density in cancer samples

Agilent ID	Gene name	FDR (%)
A_32_P171923	730402	0.00
A_32_P480177	TNN	0.00
A_23_P200298	AGL	0.00
A_24_P87036	TMEM16A	0.00
A_23_P312150	EDN2	14.87
A_23_P83388	EPPK1	14.87
A_32_P60065	F2RL2	19.82
A_32_P158272	MRNA	19.82
A_23_P105012	HRASLS2	19.82

FDR, false discovery rate.

### Genetic polymorphisms

In order to identify genetic determinants of the expression of the *UGT *genes found to be associated with MD, we performed eQTL analyses of SNPs in these genes as available from an array based GWAS study and a candidate gene study. Twenty one SNPs in *UGT *genes were present on the 109 K array from Illumina, and 9 SNPs from the candidate gene analysis. Of these, 5 SNPs were associated with the expression of *UGT *genes or other genes in their vicinity at *P *= 0.05 [see Additional file 4]. Two of these SNPs, both located in *UGT2B10 *(rs1828705, rs1828705), were significantly associated with gene expression of another *UGT *gene (*UGT2B7 *and *UGT2B28*).

## Discussion

Previously, whole genome expression profiling of normal breast tissue (all cell types included) has been performed to a limited extent [27,28]. Yang et al recently performed a study of cancer-free breast tissue obtained from mastectomies in breast cancer patients with high and low MD [29]. They identified a list of 73 genes differentially expressed between high and low MD samples. Specifically, this included the down-regulation of several transforming growth factor (TGF) β-related genes in samples with high MD. In the present study we analysed breast biopsies from 79 healthy women and tumours of 64 women with breast cancer. Twenty-four genes were differentially expressed according to MD in the healthy samples. In breast tumours, none of these 24 genes were found differentially expressed according to MD. Tumour-specific deregulation of a large number of mRNA transcripts may be expected to overshadow the MD signature. In addition, the sample size is limited and the two sample sets (cases and controls) are not directly comparable with respect to MD [see Figure S5 in Additional file 2].

In our study, three *UGT *genes (*UGT2B11, UGT2B10 *and *UGT2B7*) were differentially expressed according to MD in the breasts of healthy women. All these three enzymes had decreased expression in dense breasts. Previous knowledge links the UGT enzymes to the metabolism of female hormones known to influence the mammary glands (Figure 1). The over-representation of *UGT *genes on the list of significant genes along with a biological link makes these genes particularly interesting. In a linear regression model with age as a confounding factor, BMI and one of two probes for UGT2B10 were the only significant variables independently predicting MD, with ESR1 as a borderline significant covariate. The expression of these three *UGT2B *genes is highly correlated to each other and as expected only one probe remained in the regression model as an independent predictor of MD. BMI is known to be the strongest and most consistent epidemiological predictor of MD, and is expected to remain in the model. It is noteworthy that one of the *UGT *genes has an independent predictive value of a greater significance and magnitude than BMI. MD is determined by multiple factors. In a study of limited sample size, we can only expect to identify the strongest predictors.

UGT2B7 is postulated to protect the breast tissue from oestrogen metabolites locally [30], and this is consistent with our findings that breasts with higher MD have reduced expression of this gene. The main metabolites of oestradiol and oestrone (hydroxyl- and methoxy-oestrogen compounds) bind to the oestrogen receptor, but with a reduced affinity compared with oestradiol. UGT2B10 and 11 are not yet reported to be associated with MD or breast cancer, but UGT2B10 is involved in the metabolism of tobacco-related nitrosamines [31]. Less is known about UGT2B11. The different *UGT2B *genes are located close to each other on chromosome 4 and there is great homology between the genes [see Figure S6 in Additional file 2]. UGT1A1, previously linked to MD and breast cancer [32], is not represented on the microarray used in this study.

We have identified a set of genes differentially expressed according to MD. Interestingly, the *UGT *genes seem, to a greater extent than the other genes, to be more similarly expressed between tumour samples and normal samples from breasts with high MD as compared with normal samples from breasts with low MD [see Table S4 and Figure S7 in Additional file 2]. The other differentially expressed genes generally express the same levels in the tumours and in the biopsies from the healthy women with low MD. We cannot exclude that the *UGT *genes confer risk for breast cancer development through increasing MD, but further studies would be needed to investigate this.

We found the *UGT *genes to be differentially expressed in young women and women over 50 years of age currently on hormone therapy. SAM analysis of MD in women younger than 50 years did not give any differentially expressed genes with an FDR of less than 25%. However, several UGT-probes are on the top of the list of genes down-regulated in samples from breasts with high MD. The lack of significance could be due to low sample size (n = 30). As UGT enzymes conjugate oestradiol metabolites, its effect will be greater when there is an increased level of oestradiol present, whether the oestradiol is endogenous or exogenous. The linear regression analysis showed that UGT2B10 was predicting MD independent of age in all women, younger women and women older than 50 years currently using hormones. This leads to the hypothesis that decreased UGT expression in the breast of a woman with increased levels of female hormones confers an increased MD and possibly an increased risk of breast cancer.

The biology in breasts with high and low MD may differ, partly due to differences in proportion of fatty tissue. Therefore, we looked for differentially expressed genes in a subset of samples including only samples from breasts with MD of more than 20%. The fact that the *UGT2B *gene family is so strongly represented among the down-regulated genes (six probes representing five different *UGT2B *genes are the only genes differentially expressed with an FDR < 10E-5) indicate that reduced UGT expression is of greater significance in breasts with higher MD and lower content of fatty tissue.

We find that *ESR1 *is down-regulated in biopsies from healthy women with high MD compared with those with low MD. This is not consistent with previous findings [33] and contrary to what one would expect because *ESR1 *induces transcription and epithelial growth and high MD may contain increased amounts of epithelial cells [34,35]. However, increased levels of oestradiol have been shown to decrease levels of ESR1 in breast cancer [36], and in normal breast tissue in monkeys [37] and in mice [38]. Increased levels of oestradiol may increase MD. Elevated expression of ESR1 is common postmenopausally [37] and represents non-proliferating cells. The association between reduced levels of ESR1 and high MD may reflect high levels of oestradiol. We found that ESR1 was only a borderline significant predictor of MD in models with stepwise exclusion of covariates. In a model including ESR1 with only age or age and UGT2B10, ESR1 was significantly predicting MD. The independent contribution of ESR1 in predicting MD was significant in older women, where the effect of UGT2B10 was not present. There could be a link between UGT-expression and ESR1-expression in that reduced metabolism of oestradiol-metabolites increases the levels of ESR1-ligands (oestradiol metabolites) and hence reduces ESR1-levels. The UGT-enzyme activity may be the cause of the alterations leading to increased MD by this mechanism. Reduced ESR1 is only borderline significant in predicting MD and could also be an intermediate factor.

MD is the result of complex biological processes without any single determining factor. BMI is the single most important factor found to date, and is also significant in this study. Age seems to have its effect mainly through hormonal influence, except for in postmenopausal women not taking hormones, where age has a significant, independent effect on MD. MD is not significantly different between the two main clusters from unsupervised hierarchical clustering of the samples from healthy women. MD is hence not related to the main variation in the normal samples.

The genes whose expression we have found to be associated with MD do have a fairly high FDR in a SAM analysis and are not significant in all stratified analyses, suggesting that they may play a role in only subsets of individuals and other factors also have a significant contribution. Despite this, in linear regression models UGT2B10 is an independent predictor of MD along with BMI.

There is a substantial heritable proportion of MD. SNPs in *UGT *genes with influence on the UGT expression have been described [8,39]. We identified two UGT-SNPs associated with the expression of other *UGT *genes. Due to their homology and co-localisation on the chromosome, they may share common control loci that affect the expression of multiple *UGT *genes. It remains to be investigated in larger and better powered epidemiological studies whether any of these SNPs are associated to MD *per se*.

We do not know enough about the variability of gene expression within normal breasts to know if the genes relevant for MD are adequately represented by one biopsy taken from an area with some MD. It is previously shown that two biopsies from the same breast tumour, before and after chemotherapy, cluster together [40]. The tumours may, however, be more homogenous than normal breast tissue. Variability in gene expression within each breast will make it difficult to detect genes with only a minor influence on MD so that only the strongest factors are identified. In an unpublished dataset we found no significant difference between UGT-expression in tumours and normal adjacent tissue tested by paired t-test [see Table S5 in Additional file 2]. This is merely an indication that the expression in one breast might be similar for different locations in the breast and hence be used to look for associations with MD.

In this study, healthy individuals had higher MD than the breast cancer patients. The women recruited in the study had been referred to a breast diagnostic centre for a second look. As high MD confers an increased risk for breast cancer and mammograms with high MD are more difficult to interpret, they most likely had a higher MD. In addition, the inclusion criterion of some visible MD for biopsy may have influenced the mean MD of the study population. The two populations are not directly comparable with respect to MD and related parameters. This lack of comparability on MD does not affect the analyses of gene expression among the healthy women only.

We obtained good quality microarrays from only 79 of 120 healthy women and from 64 of 66 breast cancer patients. This was due to low mRNA-yield or low mRNA-quality. The biopsies from healthy women consistently yielded less mRNA than the tumour samples. There is significantly higher MD in the breasts of healthy women with successful microarrays than in those with unsuccessful microarrays (37% vs 29%, *P *= 0.03). As samples from breasts with low MD are under-represented in the microarray study, it is more difficult to identify genes that are differentially expressed between breast tissue with high and low MD. Despite these limitations, we have identified differentially expressed genes. These genes might have a greater significance than shown in this study.

Normal breast tissue yields less RNA than tumour tissue. The biopsies in this study were small and in agreement with the pathologist, all tissue from normal breasts was prioritised for RNA-extraction rather than histological evaluation. Imprint was not in routine use in the hospitals where we started this study. In order to make it possible for the staff to include women in this study in a busy schedule we had to use procedures already established. We do therefore not have any information about the cell types of the normal biopsies. Knowledge about the cell types present in the biopsies would have facilitated the analysis.

The two UGT2B10-probes behave differently in our dataset. Both probes map to the 3'end of the UGT2B10-gene by BLAT (98.4% homology for A_23_P7342 and 100% homology for A_24_P521559). The discrepancy in UGT2B10-expression detected by the two probes may be due to the fact that they both also share substantial sequence homology with other, but different UGT2B-genes.

## Conclusions

We have identified a set of genes that are differentially expressed according to MD in breast samples from healthy women. Some of these genes are known to influence MD and breast cancer, such as *ESR1 *and *UGT2B7*. Two less described *UGT *genes, *UGT2B10 *and *UGT2B11*, are also differentially expressed. The expression of the three *UGT *genes is reduced in samples with high MD and also in tumour samples, but does not vary between different tumour subtypes or risk groups. The UGT enzymes are known to conjugate active oestrogen-metabolites. We show that UGT2B10 expression and BMI are independent predictors of MD. The influence of reduced UGT expression was strongest in women under exposure of female hormones. Two candidate SNPs are associated with the *UGT *gene expression *in cis*. We hypothesise that reduced expression of *UGT *genes in women exposed to female sex hormones, increase MD and that this may be associated with an increased risk of breast cancer. Further studies of these genes are needed to test the hypothesis that the gene products from these genes protect the breast from the oestrogen-induced MD and thereby reducing the risk of breast cancer.

## Abbreviations

ANOVA: analysis of variance; BMI: body mass index; eQTL: expression quantitative trait loci; ESR1: oestrogen receptor; FDR: false discovery rate; GWAS: genome wide association studies; HER: human epidermal growth receptor; MD: mammographic density; SAM: significance analysis of microarrays; SNP: single nucleotide polymorphism; UGT: uridine 5'-diphospho-glucuronosyltransferase.

## Competing interests

The authors declare that they have no competing interests.

## Authors' contributions

The trial was designed by ALBD, ÅH, GU, MMH and VNK. ALBD and ÅH ensured funding. MMH, JOF, DN, LR, IKB and VDH assisted in data collection. MB and VNK are responsible for SNP analyses. VDH and TL contributed to the laboratory work. GU estimated the amount of mammographic density. OCL, VDH, HKS and MB performed statistical analyses of the data. ÅH, ALBD and VDH interpreted the results and wrote the paper. All authors were involved in reviewing the report.

## Supplementary Material

Additional file 1 **Healthy SAM MD**. Significance analysis of microarrays (SAM) for genes differentially expressed according to mammographic density (MD). Click here for file

Additional file 2 **Figures and tables**. A collection of figures and tables describing the data set and the uridine 5'-diphospho-glucuronosyltransferase (UGT) genes. The main text refers to individual figures and tables in this file. Click here for file

Additional file 3 **Healthy SAM MD stratified**. Significance analysis of microarrays (SAM) for genes differentially expressed according to mammographic density (MD) stratified on age and use of hormone therapy. Click here for file

Additional file 4 **eQTL**. Expression quantitative trait loci (eQTL) analysis of single nucleotide polymorphism (SNPs) affecting the expression of uridine 5'-diphospho-glucuronosyltransferase (UGT) genes *in cis*. Click here for file
