# Supplementary material for: Expression levels of uridine 5'-diphospho-glucuronosyltransferase genes in breast tissue from healthy women are associated with mammographic density
Source: Breast Cancer Res. 2010 Aug 27;12(4):R65. doi: 10.1186/bcr2632 (PMC2949660; doi:10.1186/bcr2632)
Supplement: Additional file 2 — Figures and tables. A collection of figures and tables describing the data set and the uridine 5'-diphospho-glucuronosyltransferase (UGT) genes. The main text refers to individual figures and tables in this file. [file bcr2632-S2.pdf]

Additional figures and tables

Figure S1: The percentage of samples with low expression of *UGT* genes (<-0.5) within tumour samples and healthy women with high (≥30%) or low (<30%) MD.

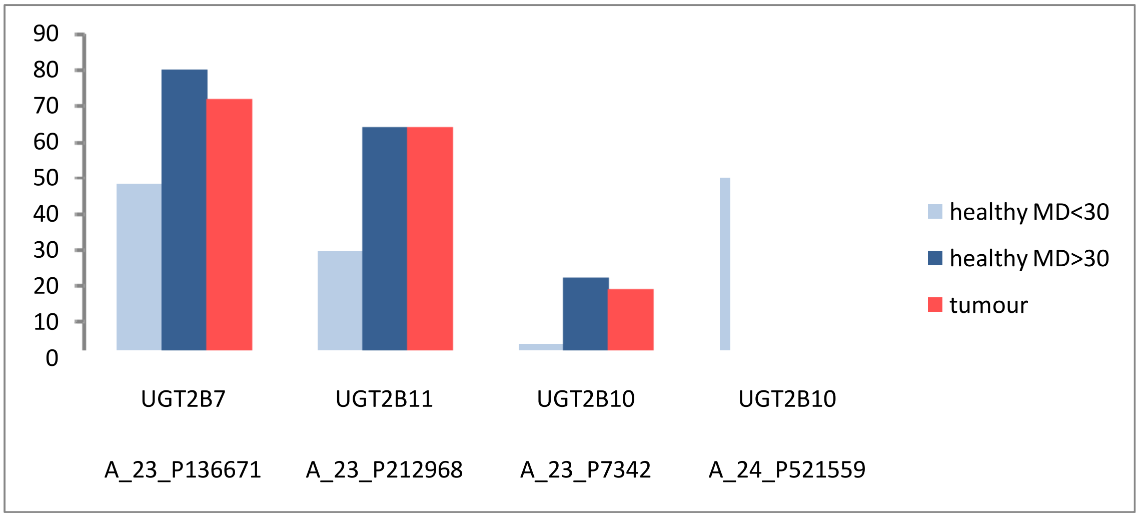

Figure S2: Clustering of the genes significantly down regulated in high MD samples. The three UGT genes cluster separately and tightly together. Samples are sorted according to MD.

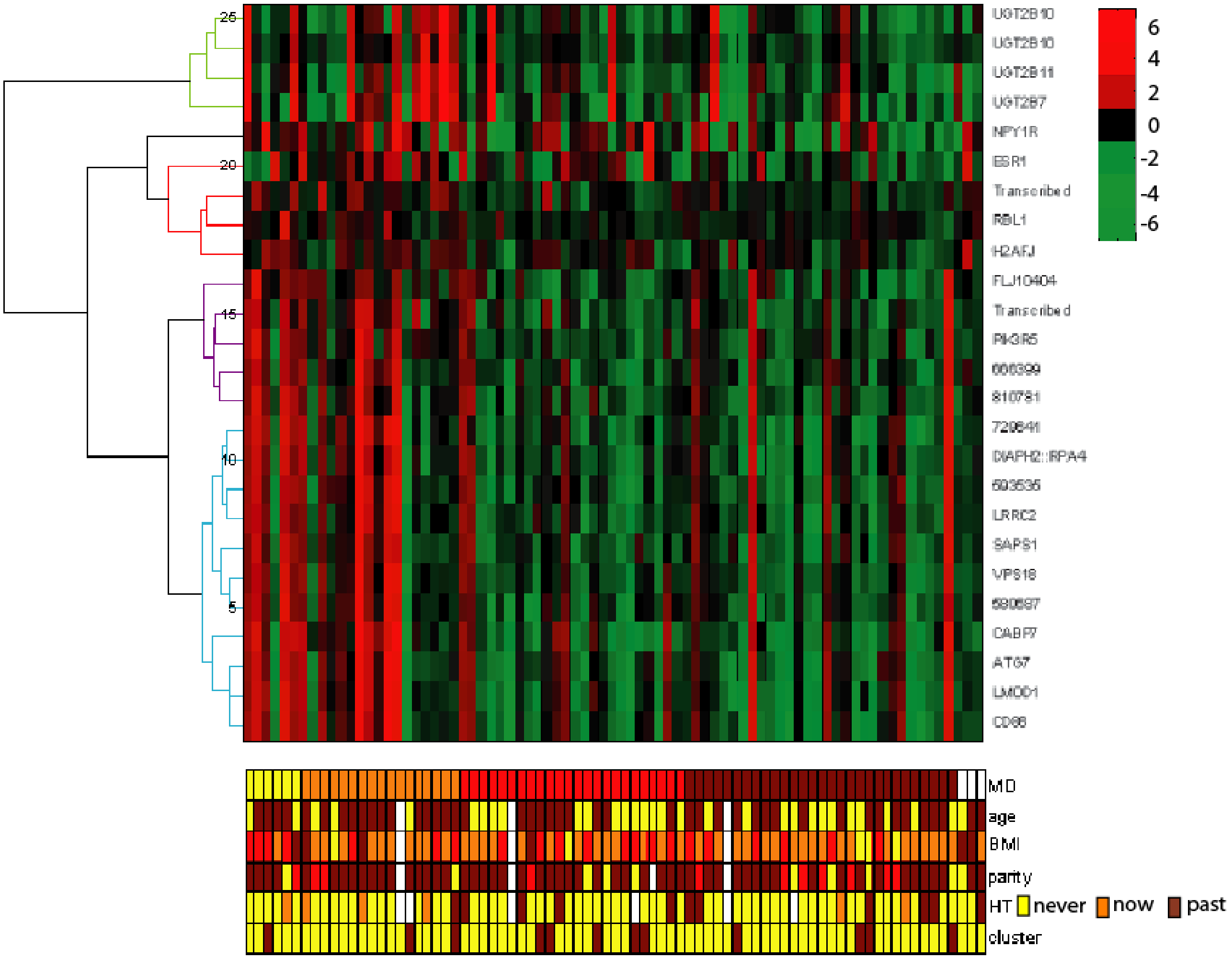

Figure S3: The expression of the different *UGT* genes is highly correlated. Expression of the four probes representing UGT-transcripts (y-axis) for each sample (x-axis) for A) healthy women and B) breast cancer patients respectively

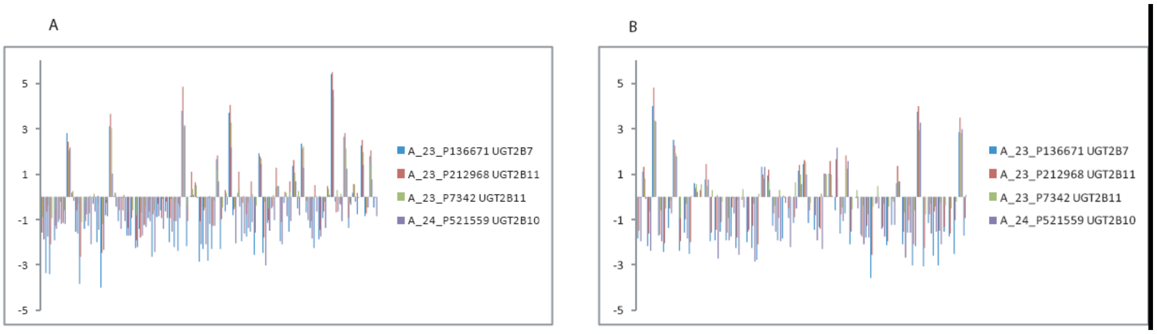

Table S1: Correlation between the expression of different UGT genes

	A_23_P136671 UGT2B7	A_23_P212968 UGT2B11	A_23_P7342 UGT2B10	A_24_P521559 UGT2B10
A_23_P136671 UGT2B7	-	0.92	0.91	0.90
A_23_P212968 UGT2B11	0.92	-	0.93	0.94
A_23_P7342 UGT2B10	0.91	0.93	-	0.90
A_24_P521559 UGT2B10	0.90	0.94	0.90	-

Table S2: MD and expression of UGT genes in samples from healthy women

		MD	A_23_P136671 UGT2B7 expression	A_23_P7342 UGT2B10 expression	A_24_P521559 UGT2B10 expression	A_23_P212968 UGT2B11 expression
age	mean <50 (n=31)	40.4	-0.83	0.15	-0.74	-0.07
	mean ≥50 (n=45)	35.2	-0.68	0.22	-0.70	-0.07
	p-value	0.25	0.72	0.78	0.92	0.99
BMI	mean <25 (n=49)	41.2	-0.84	0.12	-0.77	-0.11
	mean ≥25 (n=27)	30.0	-0.57	0.31	-0.63	0.02
	p-value	0.01	0.53	0.45	0.66	0.73
Age at first birth	mean <25 (n=38)	31.5	-0.66	0.25	-0.60	-0.03
	mean ≥25 (n=18)	39.0	-0.64	0.22	-0.48	0.00
	p-value	0.14	0.96	0.93	0.77	0.95
Current hormone therapy	mean yes (n=64)	46.5	-0.97	-0.12	-1.04	-0.50
	mean no (n=12)	35.6	-0.71	0.24	-0.64	0.01
	p-value	0.07	0.65	0.25	0.35	0.30

MD and expression of UGT genes in samples from healthy women in relation to epidemiological factors. All p-values are from two-sided t-tests not corrected for multiple testing.

Figure S3: Unsupervised hierarchical clustering showed two main clusters. MD was not significantly different between these two clusters.

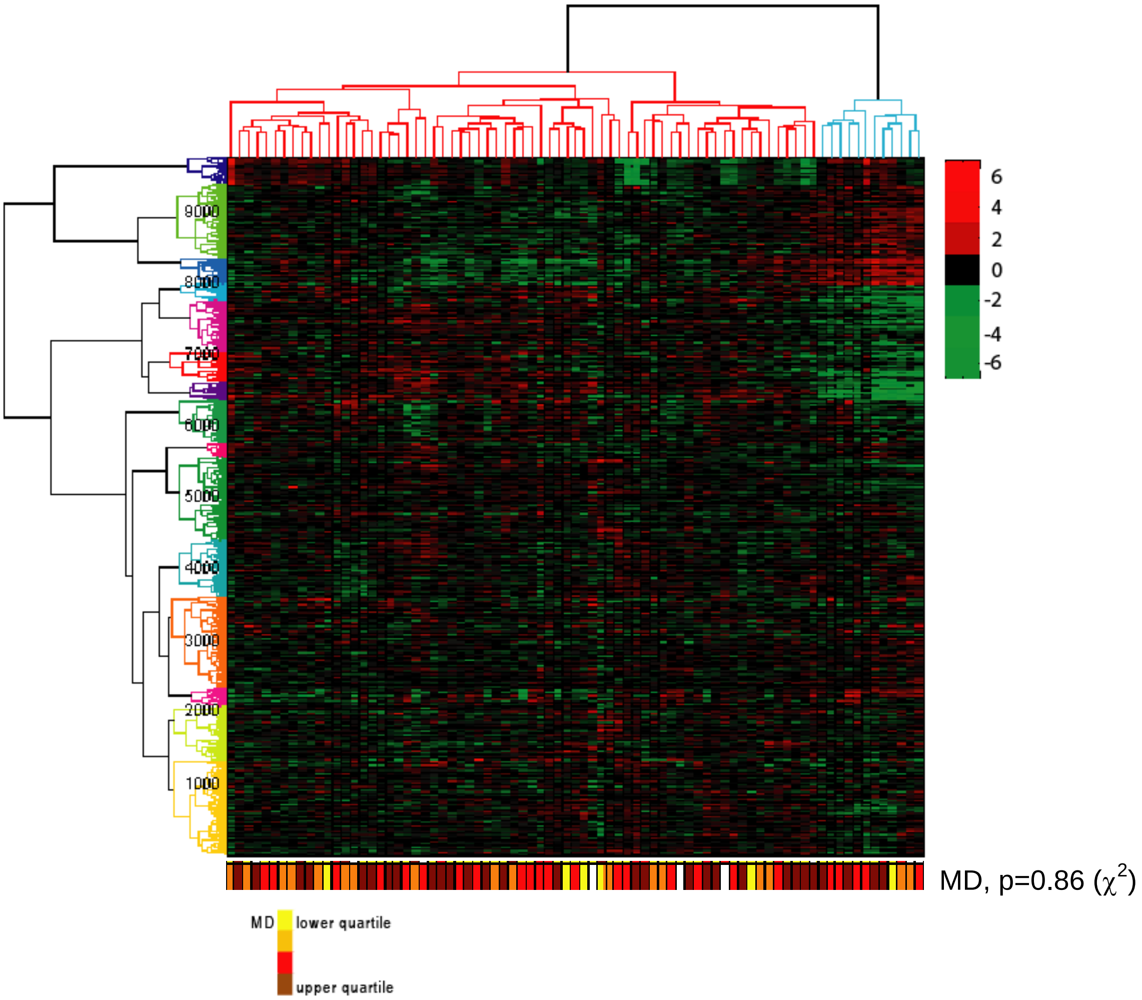

Figure S4: MD in relation to tumour subgroups

a) Boxplot of MD vs subtypes

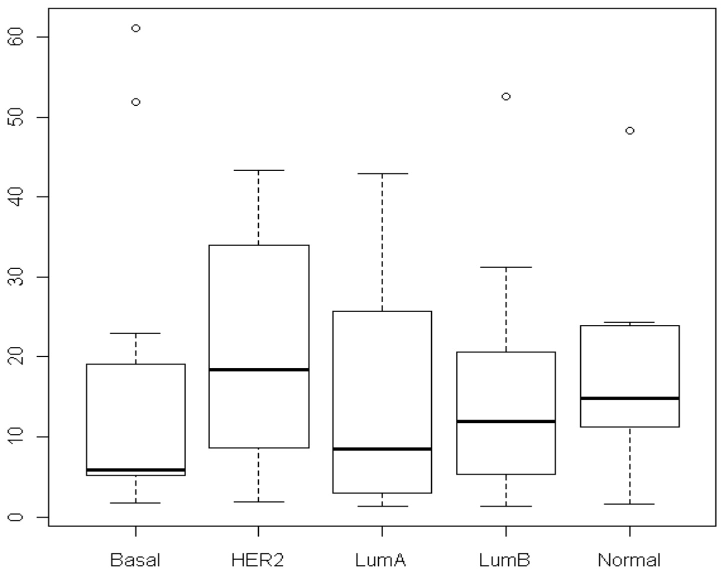

a) Boxplot of MD for each pam50 risk group (1=high, 3=medium, 5=low risk).

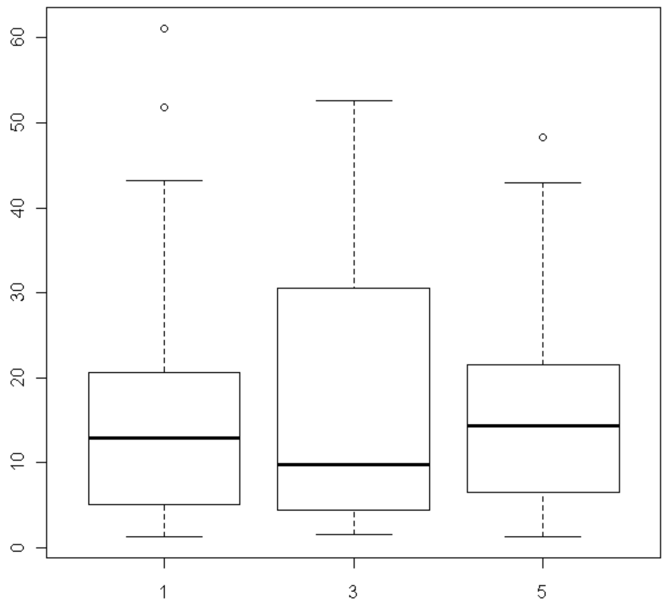

Figure S5: Distribution of MD in a) all samples (mean=), b) healthy women (mean=37%) and c) breast cancer patients (mean=16%)

a) MD frequency all women

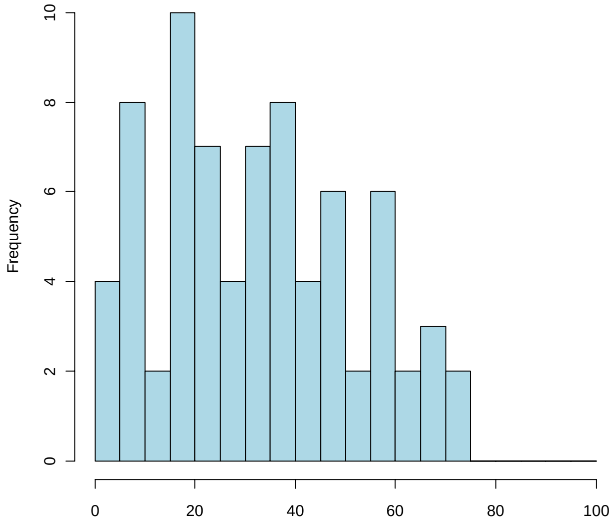

b) MD frequency healthy women

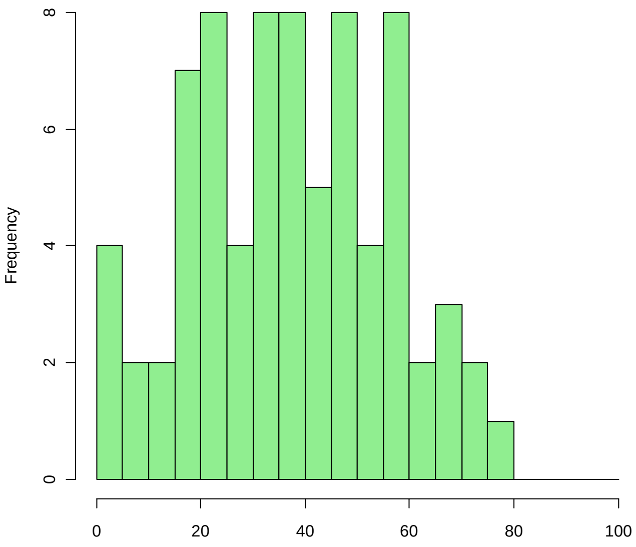

c) MD frequency breast cancer patients

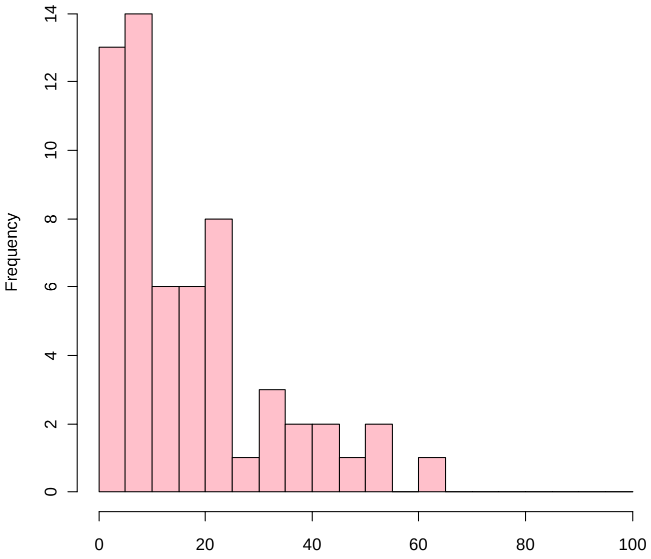

Figure S6: Localisation of UGT genes on chromosome 4:

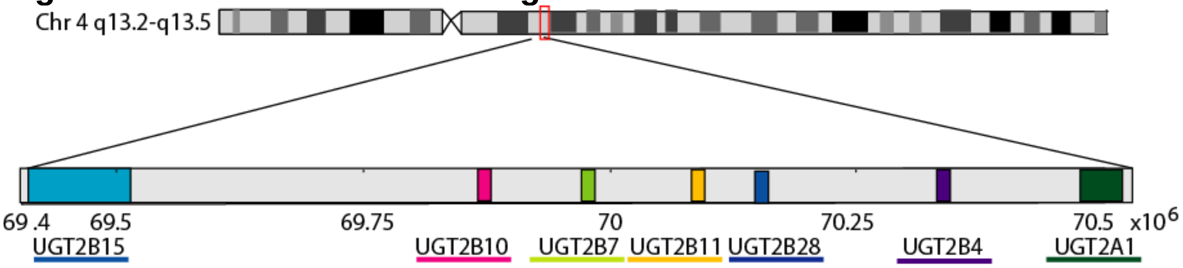

Table S4: Gene expression in samples from healthy women with differing MD compared with tumour samples.

A) MD < 30% vs MD>30%: The mean expression of all four probes representing *UGT* genes is not significantly different between tumour samples and normal samples from breasts with high MD. For three UGT probes, mean expression is significantly different between tumour samples and normal samples from breasts with low MD. This is not the case for most other genes.

Agilent ID	SYMBOL	mean t	mean n MD <30%	mean n MD>30%	ttest t vs MD<30%	ttest t vs n MD>30%
A_23_P119448	SAPS1	0.87	0.98	0.13	0.693	0.000
**A_23_P136671**	**UGT2B7**	**-1.00**	**0.21**	**-1.24**	**0.005**	**0.402**
A_23_P144244	810781	1.16	1.12	0.28	0.889	0.000
A_23_P152570	580687	1.53	1.78	0.87	0.430	0.003
A_23_P155463	LRRC2	1.39	1.60	0.65	0.478	0.001
A_23_P204277	H2AFJ	1.62	1.20	0.79	0.047	0.000
**A_23_P212968**	**UGT2B11**	**-0.43**	**0.77**	**-0.50**	**0.003**	**0.768**
A_23_P254212	DIAPH2::RPA4	1.50	1.62	0.68	0.705	0.000
A_23_P28733	RBL1	-0.90	-0.77	-1.12	0.306	0.010
A_23_P309739	ESR1	2.32	1.96	1.32	0.282	0.000
A_23_P427472	FLJ10404	1.21	1.44	0.51	0.466	0.002
A_23_P66543	PIK3R5	0.89	0.95	0.07	0.833	0.000
A_23_P69699	NPY1R	1.42	2.01	1.35	0.267	0.902
**A_23_P7342**	**UGT2B10**	**0.16**	**0.79**	**-0.13**	**0.010**	**0.054**
A_24_P131589	CD86	1.57	1.93	0.79	0.305	0.002
A_24_P177236	CABP7	1.63	1.95	0.68	0.378	0.000
A_24_P18802	VPS18	1.44	1.64	0.69	0.510	0.000
**A_24_P521559**	**UGT2B10**	**-0.70**	**0.07**	**-1.08**	**0.072**	**0.148**
A_24_P640617	Transcribed	0.97	1.04	0.18	0.801	0.000
A_24_P932736	729641	1.55	1.68	0.59	0.714	0.000
A_32_P107994	ATG7	1.51	1.78	0.74	0.413	0.001
A_32_P199824	LMOD1	1.55	1.91	0.77	0.320	0.002
A_32_P20997	Transcribed	0.25	0.56	0.10	0.008	0.092
A_32_P35668	666399	1.31	1.51	0.62	0.499	0.001
A_32_P80016	593535	1.59	1.72	0.75	0.676	0.000

Table S4 cont B) MD<20% vs MD>40%: The mean expression of all four probes representing UGT genes is not significantly different between tumour samples and normal samples from breasts with high MD, as opposed to most other probes. There is no significant difference in mean expression between tumour samples and normal samples from breasts with low MD. Contrary to most other probes, the mean expression of the UGT genes in tumours is more similar to the mean expression in normal samples from breasts with high than low MD.

			MD <20% vs >40%							
Agilent ID	SYMBOL	mean t	mean n MD <20%	mean n MD >40%	ttest t vs n MD <20%	ttest t vs n MD >40%	t-n low MD	|t-n low MD|	t-n high MD	t closer to
A_23_P119448	SAPS1	0.87	1.31	0.18	0.23	0.01	-0.44	0.44	0.69	low
**A_23_P136671**	**UGT2B7**	**-1.00**	**-0.39**	**-1.22**	**0.22**	**0.52**	**-0.61**	**0.61**	**0.21**	**high**
A_23_P144244	810781	1.16	1.46	0.37	0.42	0.00	-0.31	0.31	0.79	low
A_23_P152570	580687	1.53	2.12	0.98	0.13	0.04	-0.59	0.59	0.55	high
A_23_P155463	LRRC2	1.39	1.78	0.72	0.29	0.01	-0.39	0.39	0.67	low
A_23_P204277	H2AFJ	1.62	1.18	0.73	0.11	0.00	0.44	0.44	0.89	low
**A_23_P212968**	**UGT2B11**	**-0.43**	**0.24**	**-0.52**	**0.15**	**0.75**	**-0.66**	**0.66**	**0.09**	**high**
A_23_P254212	DIAPH2::RPA4	1.50	1.98	0.75	0.23	0.01	-0.48	0.48	0.76	low
A_23_P28733	RBL1	-0.90	-0.70	-1.15	0.18	0.01	-0.20	0.20	0.26	low
A_23_P309739	ESR1	2.32	1.89	1.21	0.32	0.00	0.43	0.43	1.12	low
A_23_P427472	FLJ10404	1.21	1.70	0.53	0.24	0.01	-0.49	0.49	0.68	low
A_23_P66543	PIK3R5	0.89	1.14	0.17	0.51	0.01	-0.25	0.25	0.72	low
A_23_P69699	NPY1R	1.42	2.21	1.26	0.25	0.73	-0.78	0.78	0.16	high
**A_23_P7342**	**UGT2B10**	**0.16**	**0.50**	**-0.15**	**0.20**	**0.07**	**-0.34**	**0.34**	**0.30**	**high**
A_24_P131589	CD86	1.57	2.26	0.87	0.10	0.02	-0.68	0.68	0.70	low
A_24_P177236	CABP7	1.63	2.25	0.77	0.17	0.01	-0.62	0.62	0.86	low
A_24_P18802	VPS18	1.44	1.88	0.77	0.23	0.01	-0.44	0.44	0.67	low
**A_24_P521559**	**UGT2B10**	**-0.70**	**-0.38**	**-1.14**	**0.52**	**0.17**	**-0.32**	**0.32**	**0.44**	**low**
A_24_P640617	Transcribed	0.97	1.25	0.32	0.42	0.01	-0.28	0.28	0.65	low
A_24_P932736	729641	1.55	2.10	0.64	0.19	0.00	-0.55	0.55	0.91	low
A_32_P107994	ATG7	1.51	2.07	0.83	0.17	0.02	-0.57	0.57	0.68	low
A_32_P199824	LMOD1	1.55	2.22	0.84	0.12	0.02	-0.67	0.67	0.71	low
A_32_P20997	Transcribed	0.25	0.69	0.15	0.00	0.28	-0.44	0.44	0.10	high
A_32_P35668	666399	1.31	1.71	0.71	0.28	0.02	-0.41	0.41	0.60	low
A_32_P80016	593535	1.59	2.02	0.82	0.26	0.00	-0.42	0.42	0.78	low

Figure S7: The Kullback-Leibler divergence between UGT expression in tumours and healthy samples with high and low MD respectively. Small divergence means more similar distribution in the two populations tested. For three of four UGT probes, the distribution in tumour samples is more similar to the distribution in healthy individuals with high-MD than with low-MD. (The first UGT2B10-probe is A_23_P7342, the probe that is significant in the GLM-analysis, the second is A_24_P521559).

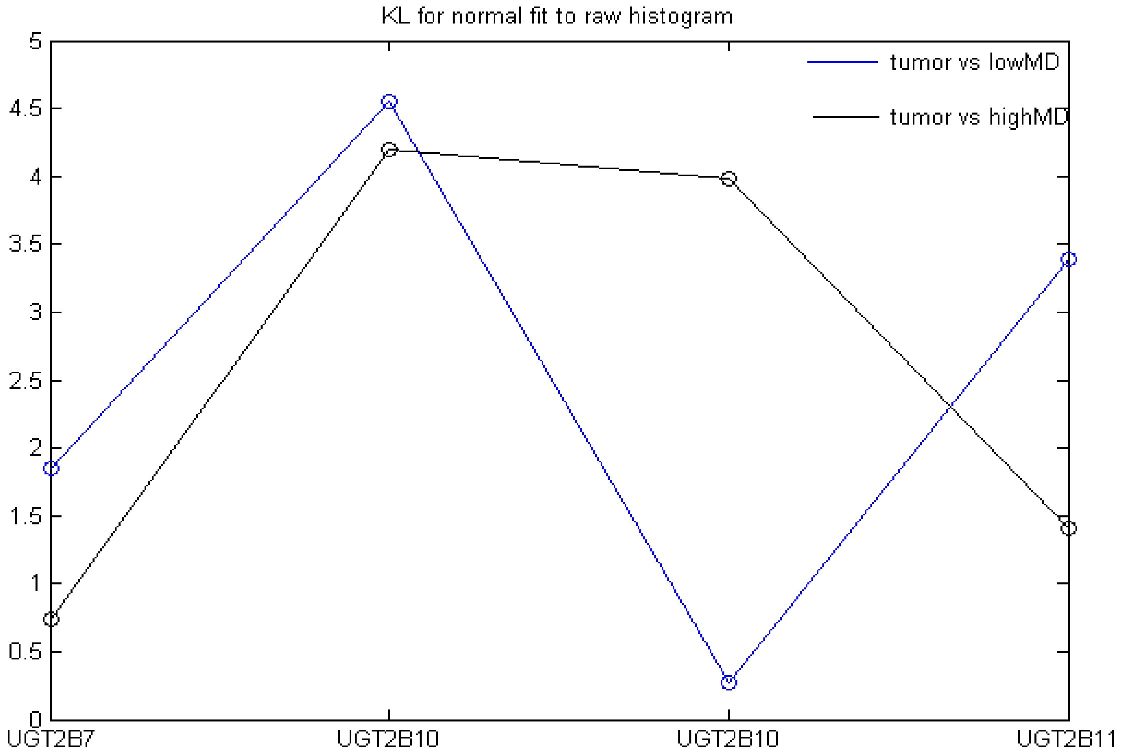

Table S5: Gene expression of UGT2B10 in tumour samples (T) and normal adjacent samples (N) from the same breast in an unpublished dataset. There is no significant difference in mean expression by pair wise t-test.

	A_23_P7342 UGT2B10		A_23_P7342 UGT2B10
CM 1N	9.75	CM 1T	7.52
CM 9N	8.62	CM 9T	8.49
CM 10N	8.77	CM 10T	7.27
CM 11N	9.26	CM 11T	6.65
CM 13N	10.32	CM 13T	7.43
CM 18N	10.69	CM 18T	8.79
CM 19N	9.12	CM 19T	9.11
CM 26N	8.78	CM 26T	7.31
CM 31N	8.00	CM 31T	8.07
CM 32N	7.73	CM 32T	7.23
CM 38N	10.96	CM 38T	7.32
CM 41N	9.07	CM 41T	11.71
CM 46N	8.74	CM 46T	11.21
CM 47N	11.08	CM 47T	8.00
CM 54N	7.80	CM 54T	13.14
CM 56N	7.92	CM 56T	17.48
CMG24N	6.66	CMG24T	13.89
CMG43N	8.24	CMG43T	10.59
CM 44N	8.89	CM 44T	7.75
average	8.97	average	9.42
p-value	0.60	(pair wise t-test)	

Table S6: Range of MD in the breasts of healthy women

**max**	**77.3133466**
**min**	**1.28417454**
**mean**	**28.1668549**
**median**	**23.5504612**

Supplemental discussion

Gene expression microarray analyses using tissue adjacent to a breast tumour have previously been done [29, 41]. The expression profile in these normal samples will be influenced by the neighbouring breast tumour [42]. Breast reduction mammoplasties have also been used in analysis of healthy breast tissue [43]. These samples are generally collected from large breasts with a higher than average proportion of fatty tissue which may also skew the analyses to some extent. Our study analysed a population more representative of the population of women at risk for developing breast cancer, since we have studied normal breast tissue from women with no malignant disease and not undergoing breast reduction mammoplasties.
